# Immunogenicity of chimeric haemagglutinin-based, universal influenza virus vaccine candidates: interim results of a randomised, placebo-controlled, phase 1 clinical trial

**DOI:** 10.1016/S1473-3099(19)30393-7

**Published:** 2019-10-17

**Authors:** David I Bernstein, Jeffrey Guptill, Abdollah Naficy, Raffael Nachbagauer, Francesco Berlanda-Scorza, Jodi Feser, Patrick C Wilson, Alicia Solórzano, Marie Van der Wielen, Emmanuel B Walter, Randy A Albrecht, Kristen N Buschle, Yao-qing Chen, Carine Claeys, Michelle Dickey, Haley L Dugan, Megan E Ermler, Debra Freeman, Min Gao, Christopher Gast, Jenna J Guthmiller, Rong Hai, Carole Henry, Linda Yu-Ling Lan, Monica McNeal, Anna-Karin E Palm, Dustin G Shaw, Christopher T Stamper, Weina Sun, Victoria Sutton, Micah E Tepora, Rahnuma Wahid, Heather Wenzel, Teddy John Wohlbold, Bruce L Innis, Adolfo García-Sastre, Peter Palese, Florian Krammer

**Affiliations:** 1 Department of Pediatrics, University of Cincinnati College of Medicine, Cincinnati, OH, USA (Prof D I Bernstein MD, K N Buschle MSN, M Dickey MSN, M McNeal MS); 2 Division of Infectious Diseases, Cincinnati Children’s Hospital Medical Center, Cincinnati, OH, USA (Prof D I Bernstein, K N Buschle, M Dickey, M McNeal); 3 Duke Early Phase Clinical Research Unit, Duke Clinical Research Institute, Durham, NC, USA (J Guptill MD, Prof E B Walter MD, D Freeman, M Gao MS, V Sutton BS); 4 Center for Vaccine Innovation and Access, PATH, Seattle, WA, USA (A Naficy MD, F Berlanda-Scorza PhD, J Feser MPH, C Gast PhD, R Wahid PhD, H Wenzel MS, B L Innis MD); 5 Department of Microbiology (R Nachbagauer MD, A Solórzano PhD, R A Albrecht PhD, M E Ermler PhD, R Hai PhD, W Sun PhD, T J Wohlbold PhD, Prof A García-Sastre PhD, Prof P Palese PhD, Prof F Krammer PhD); 6 Global Health and Emerging Pathogens Institute (R A Albrecht, Prof A García-Sastre); 7 Department of Medicine (Prof A García-Sastre, Prof P Palese); 8 Icahn School of Medicine at Mount Sinai, New York, NY, USA; Section of Rheumatology, Department of Medicine (Prof P C Wilson PhD, Y Chen PhD, J J Guthmiller PhD, C Henry PhD, A-K E Palm PhD, M E Tepora MS); 9 The Committee on Immunology (Prof P C Wilson, H L Dugan BA, L Y-L Lan DVM, D G Shaw BA,C T Stamper BS); 10 University of Chicago, Chicago, IL, USA; GlaxoSmithKline, Wavre, Belgium (M Van der Wielen MD, C Claeys MD); 11 Duke Human Vaccine Institute, Duke University School of Medicine, Durham, NC, USA (Prof E B Walter); 12 GlaxoSmithKline, Collegeville, PA, USA (M E Ermler); 13 Department of Microbiology and Plant Pathology, Institute for Integrative Genome Biology, University of California, Riverside, CA, USA (R Hai)

## Abstract

**Background:**

Influenza viruses cause substantial annual morbidity and mortality globally. Current vaccines protect against influenza only when well matched to the circulating strains. However, antigenic drift can cause considerable mismatches between vaccine and circulating strains, substantially reducing vaccine effectiveness. Moreover, current seasonal vaccines are ineffective against pandemic influenza, and production of a vaccine matched to a newly emerging virus strain takes months. Therefore, there is an unmet medical need for a broadly protective influenza virus vaccine. We aimed to test the ability of chimeric H1 haemagglutinin-based universal influenza virus vaccine candidates to induce broadly cross-reactive antibodies targeting the stalk domain of group 1 haemagglutinin-expressing influenza viruses.

**Methods:**

We did a randomised, observer-blinded, phase 1 study in healthy adults in two centres in the USA. Participants were randomly assigned to one of three prime–boost, chimeric haemagglutinin-based vaccine regimens or one of two placebo groups. The vaccine regimens included a chimeric H8/1, intranasal, live-attenuated vaccine on day 1 followed by a non-adjuvanted, chimeric H5/1, intramuscular, inactivated vaccine on day 85; the same regimen but with the inactivated vaccine being adjuvanted with AS03; and an AS03-adjuvanted, chimeric H8/1, intramuscular, inactivated vaccine followed by an AS03-adjuvanted, chimeric H5/1, intramuscular, inactivated vaccine. In this planned interim analysis, the primary endpoints of reactogenicity and safety were assessed by blinded study group. We also assessed anti-H1 haemagglutinin stalk, anti-H2, anti-H9, and anti-H18 IgG antibody titres and plasmablast and memory B-cell responses in peripheral blood. This trial is registered with ClinicalTrials.gov, number NCT03300050.

**Findings:**

Between Oct 10, 2017, and Nov 27, 2017, 65 participants were enrolled and randomly assigned. The adjuvanted inactivated vaccine, but not the live-attenuated vaccine, induced a substantial serum IgG antibody response after the prime immunisation, with a seven times increase in anti-H1 stalk antibody titres on day 29. After boost immunisation, all vaccine regimens induced detectable anti-H1 stalk antibody (2·2–5·6 times induction over baseline), cross-reactive serum IgG antibody, and peripheral blood plasmablast responses. An unsolicited adverse event was reported for 29 (48%) of 61 participants. Solicited local adverse events were reported in 12 (48%) of 25 participants following prime vaccination with intramuscular study product or placebo, in 12 (33%) of 36 after prime immunisation with intranasal study product or placebo, and in 18 (32%) of 56 following booster doses of study product or placebo. Solicited systemic adverse events were reported in 14 (56%) of 25 after prime immunisation with intramuscular study product or placebo, in 22 (61%) of 36 after immunisation with intranasal study product or placebo, and in 21 (38%) of 56 after booster doses of study product or placebo. Disaggregated safety data were not available at the time of this interim analysis.

**Interpretation:**

The tested chimeric haemagglutinin-based, universal influenza virus vaccine regimens elicited cross-reactive serum IgG antibodies that targeted the conserved haemagglutinin stalk domain. This is the first proof-of-principle study to show that high anti-stalk titres can be induced by a rationally designed vaccine in humans and opens up avenues for further development of universal influenza virus vaccines. On the basis of the blinded study group, the vaccine regimens were tolerable and no safety concerns were observed.

**Funding:**

Bill & Melinda Gates Foundation.

## Introduction

Seasonal influenza viruses cause up to 650 000 deaths and 3–5 million severe infections annually worldwide.^1^ Current vaccines protect well against influenza when they match circulating strains, but must be updated and re-administered annually because of antigenic drift of the virus. Annual strain selection for seasonal vaccines is based on predictions, therefore mismatches often occur, leading to a substantial decrease in vaccine effectiveness. Additionally, pandemics occur in irregular intervals causing substantial morbidity and mortality. Matched vaccines have to be manufactured for these emerging viruses, a process that takes about 6 months,^2^ during which time the population remains vulnerable. A vaccine that protects against influenza independently of antigenic drift or shift is, therefore, urgently needed, as emphasised by the National Institute of Allergy and Infectious Diseases.^3–5^

**Research in context**

**Evidence before this study**

PubMed was searched with the terms “universal influenza virus vaccine”, “hemagglutinin stalk”, “influenza heterosubtypic immunity”, and “anti-stalk antibody”, without language restrictions, for literature published between database inception and March 21, 2019. The first paper regarding a broadly protective haemagglutinin stalk-reactive antibody was published in 1993; similar antibodies were then discovered for the first time in humans in 2008. The existence of these antibodies in humans suggested that designing a universal influenza virus vaccine might be feasible. Several haemagglutinin stalk-based vaccines have been preclinically tested since then, including the chimeric haemagglutinin candidates evaluated in this study. Preclinical experiments with chimeric haemagglutinin constructs showed that priming with a live-attenuated virus vaccine followed by boosting with an inactivated, adjuvanted virus vaccine (both expressing chimeric haemagglutinins with the same stalk but different head domains) provided excellent protection. This finding formed the basis of the phase 1 study reported here. Several clinical trials with broadly protective influenza virus vaccine candidates based on internal viral proteins or peptides that trigger T-cell responses have been reported, but no clinical data exist for stalk-based vaccine approaches. As emphasised by the US National Institutes of Health, the Bill & Melinda Gates Foundation, and European funding agencies, improved and preferentially universal influenza virus vaccines are urgently needed.

**Added value of this study**

This is the first study to report testing of a haemagglutinin stalk-based universal influenza virus vaccine in humans. The different vaccination regimens tested induced high titres of anti-haemagglutinin stalk serum IgG antibodies, but the strength of induction varied between regimens. The induction was strongest with inactivated, AS03-adjuvanted vaccines. The induced antibodies, independently of the vaccination regimen, bound broadly to heterosubtypic group 1 influenza virus haemagglutinins, including H2, H9, and H18. Our data suggest that rationally designed antigens and vaccine regimens can induce stalk-directed heterosubtypic antibodies in adults and pave the way for further clinical development of our vaccine candidates.

**Implications of all available evidence**

This study provides evidence that group 1 (eg, H1, H2, and H5) chimeric haemagglutinin-based universal influenza virus vaccines can induce high serum titres of anti-haemagglutinin stalk IgG with heterosubtypic group 1 cross-reactivity. This result suggests that group 2 (eg, H3 and H7) constructs, which are in preclinical development, might induce similar immune responses. In more general terms, our findings provide evidence that stalk-based immunogens can induce high titres of cross-reactive antibodies. This evidence opens up opportunities for the clinical development of urgently needed broadly protective or universal influenza virus vaccines that might provide protection from drifted seasonal, zoonotic, and emerging pandemic influenza virus infection.

The majority of the antibody response induced by seasonal vaccines is directed towards the immunodominant head domain of haemagglutinin, which has high plasticity and is responsible for most of the antigenic drift.^6,7^ The membrane proximal stalk domain of haemagglutinin is highly conserved but immuno-subdominant, and it is not readily targeted by the immune system. However, both memory B cells targeting the stalk and low anti-stalk antibody titres have been detected in adults.^8–10^ Monoclonal haemagglutinin stalk-specific antibodies isolated from mice and humans show broad in-vitro neutralisation of influenza virus subtypes (but no haemagglutination-inhibiting activity), usually restricted to one of the two types of influenza A virus hemagglutinins, group 1 (H1, H2, H5, H6, H8, H9, H11, H12, H13, H16, H17, and H18) or group 2 (H3, H4, H7, H10, H14, and H15).^11^ Passive immunisation of animals with stalk-reactive antibodies protected them from challenge with both seasonal and avian influenza virus strains and subtypes within the same haemagglutinin group.^11^

**Figure 1:**
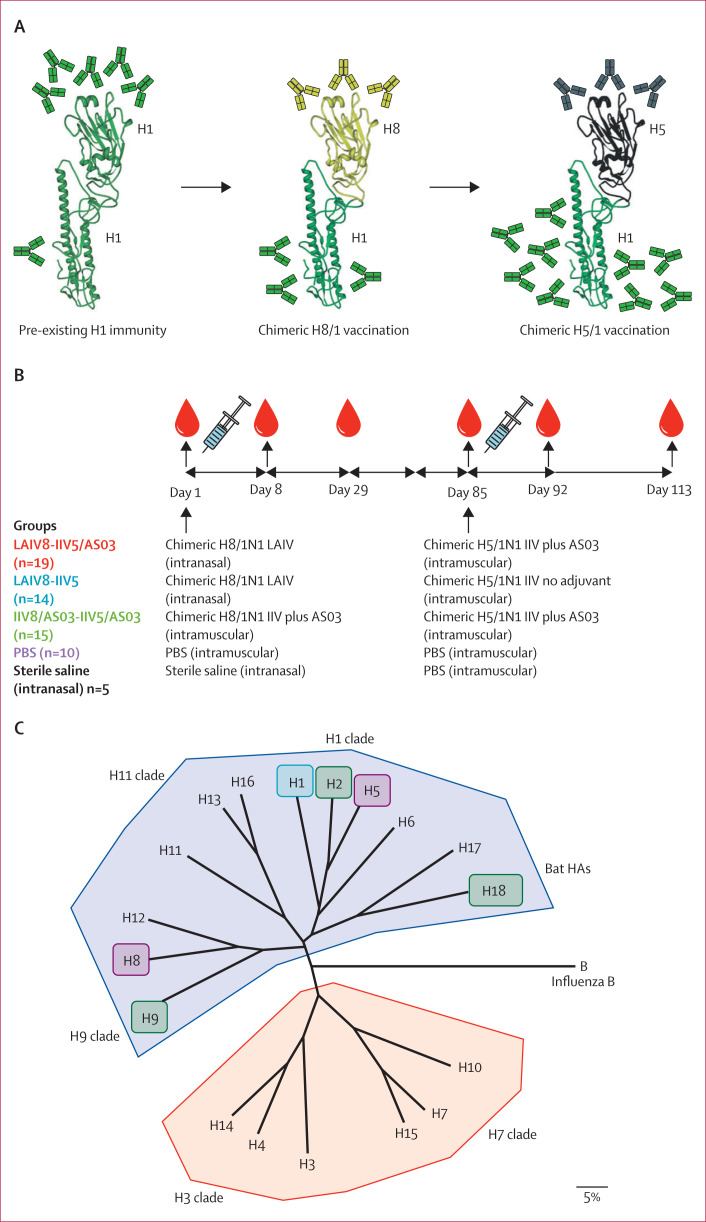
Schematic of the chimeric HA vaccination regimens and trial design (A) Vaccination strategy. Adults have pre-existing antibodies targeting both the membrane-distal head domain (top) and the membrane-proximal stalk domain (bottom) of H1 HA (green) due to previous exposure to influenza viruses. Vaccination with a chimeric H8/1 construct is expected to elicit some antibodies against the head domain (yellow), to which humans are naive, while substantially boosting H1 stalk antibodies. An additional booster vaccination with chimeric H5/1 HA was expected to provide an additional increase in antibodies targeting the HA stalk domain. Structures were adapted from RCSB Protein Data Bank ID 1RU7 and visualised in Protein Workshop.12 (B) Vaccination and blood collection schedule. (C) A phylogenetic tree based on percentage amino acid difference was constructed to illustrate the evolutionary distance of the antigens used for the ELISA analysis. The H1 (blue) stalk domain was used in the vaccines. H2 is closely related to H1, whereas H9 and H18 (all highlighted in green) are distantly related HAs within influenza A group 1. HA subtypes that donated heads to the vaccine constructs (H5 and H8) are shown in purple. Group 1 HAs are shaded in purple and group 2 in orange. HA clades are indicated within the groups. The scale bar represents a 5% difference in amino acid sequence. IIV=inactivated influenza vaccine. LAIV=live-attenuated influenza vaccine. PBS=phosphate-buffered saline. HA=haemagglutinin.

We have designed a vaccination strategy to selectively induce high concentrations of anti-stalk antibodies using sequential vaccination with chimeric haemagglutinin constructs (figure 1A). These constructs consist of H1 (group 1), H3 (group 2), or influenza B virus haemagglutinin stalks combined with head domains to which humans are naive.^13–15^ In mouse and ferret influenza vaccination and challenge models, sequential vaccination with chimeric haemagglutinins that featured the same (eg, H1) stalk but different head (eg, H5 and H8) domains induced strong anti-stalk responses, with relatively weaker responses to the respective head domains, protecting them from challenge with diverse influenza viruses.^16–19^ This response is achieved because immunity to the stalk is first primed and subsequent vaccinations lead to recall responses against the stalk, which the immune system has already seen. However, the different heads are antigenically distinct from one another and elicit only primary responses (figure 1A). With this approach, we hypothesise that a set of trivalent chimeric haemagglutinin-based vaccines directing an immune response to the H1 or group 1, H3 or group 2, and influenza B virus stalks could confer universal protection against influenza A and B. In this study, we have used monovalent H1 (group 1) chimeric haemagglutinin vaccines to test this concept.

Specifically, we tested whether stalk antibodies in adults with pre-existing immunity to influenza virus haemagglutinin^8^ could be boosted using chimeric H8/1 and H5/1 constructs (figure 1A). Two approaches were tested. The first included an initial vaccination with a live-attenuated influenza virus vaccine (LAIV) expressing chimeric H8/1 and neuraminidase subtype 1 (N1) followed by a chimeric H5/1N1 split virion inactivated influenza virus vaccine (IIV) boost given with or without AS03 adjuvant.^20^ The second approach included vaccination with AS03-adjuvanted chimeric H8/1N1 IIV followed by a boost with AS03-adjuvanted chimeric H5/1N1 IIV. The heterologous prime–boost regimen comprised of the LAIV followed by the IIV was tested because it was previously shown to induce optimal antibody responses with avian or pre-pandemic influenza virus vaccines in humans and non-human primates and with chimeric haemagglutinin-based vaccines in ferrets.^18,21–24^ In studies in ferrets,^18,25^ we observed that the chimeric haemagglutinin-based LAIV–IIV regimen induced protection against infection that was superior to the protection observed with chimeric haemagglutinin IIV–IIV regimens. This observation led to the hypothesis tested in this trial that administration of a chimeric haemagglutinin-based LAIV through the intranasal route as the first dose in a prime–boost immunisation sequence, followed by a chimeric haemagglutinin-based IIV boost via the intramuscular route, might offer superior immunogenicity compared with two intramuscular doses of the chimeric IIV.

## Methods

### Study design and participants

We did a randomised, placebo-controlled, observer-blinded, phase 1 study at Cincinnati Children’s Hospital Medical Center (Cincinnati, OH, USA) and the Duke Early Phase Clinical Research Unit (Durham, NC, USA), with participants recruited from the community. Enrolment began on Oct 10, 2017, and participants remained in follow-up until Aug 9, 2019.

Healthy men or non-pregnant women aged 18–39 years who were either of non-childbearing potential, sterile, or willing to practice adequate contraception from the first vaccination to day 85 of the study were included. Beyond the general exclusion criteria for an investigational study in healthy adults, additional exclusion criteria included diagnosis of a deviated nasal septum or nasal obstruction, recommendation for annual vaccination against influenza or living with or caring for people at high risk of influenza-related complications, history of influenza vaccination within 6 months before study enrolment or unwillingness to forego seasonal influenza vaccination, history of vaccination with an investigational pandemic influenza vaccine other than an H1N1pdm09 vaccine, history of excessive daytime sleepiness or narcolepsy, history of Guillian-Barré syndrome, history of anaphylactic-type reaction to consumption of eggs, or history of severe adverse reaction to a previous influenza vaccine.

### Randomisation and masking

Participants were randomly assigned 4:3:1:3:2 to the LAIV8-IIV5/AS03, LAIV8-IIV5, phosphate-buffered saline (PBS) placebo, IIV8/AS03-IIV5/AS03, or sterile saline placebo groups (figure 1B). Randomisation was blocked (block size 13) and stratified by site. The randomisation sequence was generated independently by a contract research organisation, Emmes Corporation. The study nurse enrolled participants and assessed eligibility, and the data management system randomly assigned participants to groups following entry of data regarding eligibility by the study nurse. Eligible participants were entered into a dosing cohort within an external data management system, which provided a coded treatment number. The number was printed and taken to the pharmacist, who was unmasked to group assignment. The pharmacist matched the coded treatment numbers with the corresponding treatment assignments and prepared the study product, which they then masked in an opaque plastic sleeve. This was an observer-blind study: the participants and study staff involved in clinical evaluation of the participants were masked to group assignment.

### Procedures

The chimeric H8/1N1 LAIV, based on the A/Leningrad/134/17/57 backbone,^26^ and the chimeric H5/1N1 and chimeric H8/1N1 IIV vaccine constructs are described in detail elsewhere.^18^ The LAIV was manufactured in embryonated chicken eggs by Meridian Life Science (Memphis, TN, USA) and administered intranasally as drops at a dose of 10^7·5^ (plus or minus ^0·5^) 50% egg infectious dose, formulated in a total volume of 0·5 mL sterile saline. In more detail, the LAIV was delivered using a 1 mL syringe without needle. The vaccinee was requested to blow their nose before administration and to lay supine with their head tilted backward. Half of the dose (0·25 mL) was then administered dropwise into one nostril by placing the syringe at the entrance of the nostril and slowly pressing the plunger of the syringe, pointing straight up the vaccinee’s nasal cavity. The remaining half of the dose (0·25 mL) was administered to the second nostril. The vaccinee was asked not to sneeze during administration and was requested to remain with their head tilted back for 5 min after administration.

The chimeric H5/1N1 and H8/1N1 IIVs were produced as split vaccines by GlaxoSmithKline (Wavre, Belgium), similar to the vaccines previously described.^18,27^ The IIVs were administered intramuscularly at a dose of 15 μg of haemagglutinin in a volume of 0·5 mL of PBS or AS03 as adjuvant.

The three vaccine regimens tested were (figure 1B): chimeric H8/1N1 LAIV followed by AS03-adjuvanted chimeric H5/1N1 IIV (LAIV8-IIV5/AS03 group), chimeric H8/1N1 LAIV followed by non-adjuvanted chimeric H5/1N1 IIV (LAIV8-IIV5 group), and AS03-adjuvanted chimeric H8/1N1 IIV followed by AS03-adjuvanted chimeric H5/1N1 IIV (IIV8/AS03-IIV5/AS03 group). The first placebo group received 0·5 mL of PBS intramuscularly as prime and booster doses (PBS group). The second group received 0·5 mL sterile saline intranasally followed by an intramuscular injection of 0·5 mL PBS (sterile saline group). Individuals who received the LAIV or intranasal sterile saline as the prime dose were confined to a containment unit for at least 5 days after vaccine administration and until no shedding of the vaccine virus was detected in three consecutive real-time PCR (rtPCR) assays of nasal or oropharyngeal swabs collected over 48 h. The cutoff for rtPCR assays was cycle threshold value of 40 or less. To maintain the observer-blind status of the study, the results of inpatient daily influenza A virus tests were provided only to a designated unmasked member of the study team. This person communicated positive results to the masked investigators and study team members on a need to know basis (ie, any positive result that might delay discharge from the inpatient unit). PCR positive samples were also cultured on Mardin Darby canine kidney cells.

Vaccines and placebo treatments were administered on study days 1 (chimeric H8/1N1) and 85 (chimeric H5/H1N1). Blood samples for this interim analysis were taken on study days 1 (day –1 for participants receiving LAIV, but indicated as day 1 throughout the manuscript for simplicity), 8, 29, 85, 92, and 113 (figure 1B). In this pre-planned interim analysis, which was done before the complete set of results was available, we assessed serum IgG levels to the haemagglutinin stalk and to heterosubtypic full-length haemagglutinins, as well as plasma-blast and memory B-cell responses to the haemagglutinin stalk and full-length wild-type haemagglutinin.

Following each vaccine administration, participants were observed for at least 60 min for any immediate adverse reactions. Solicited adverse events recorded for 7 days after each vaccination included local reactions at the administration site (pain, swelling, and erythema after intramuscular administration, or rhinorrhea and nasal congestion after intranasal administration) and systemic reactions (fever, shivering, headache, fatigue, myalgia, arthralgia, cough, sore throat, wheezing, nausea, vomiting, abdominal pain, and diarrhoea). Unsolicited adverse events were recorded for 28 days after each vaccination; all medically attended adverse events, serious adverse events, potential immune-mediated diseases, influenza-like illnesses, adverse events leading to participant withdrawal from the study, and adverse events related to abnormal clinical safety laboratory tests were recorded throughout the study duration. Safety results were blinded to study group and disaggregated safety data were not available.

Quantitative serum IgG ELISAs were done according to qualified standard operating procedures by NEOMEDLABS (Laval, Canada). ELISA antigen included a chimeric H6/1 recombinant protein that features an H6 head domain (strain A/mallard/Sweden/81/02 [H6N1]) fused to the same H1 stalk domain as expressed by the vaccines (strain A/California/04/09 [H1N1]) but with a stabilising mutation.^8,28^ This substrate was used to measure antibodies to the stalk domain of H1 since humans are naive to the H6 head domain. Full-length (ecto-domain) recombinant haemagglutinin proteins subtype H2 (strain A/mallard/Netherlands/5/99 [H2N9]; stalk domain shares about 78% aminoacid identity with the stalk domain of the H1 vaccine strain), H9 (strain A/chicken/Hong Kong/G9/97 [H9N2]; stalk domain shares about 59% aminoacid identity with the stalk domain of the H1 vaccine strain), and H18 (strain A/flat-faced bat/Peru/033/10 [H18N11]; stalk domain shares about 65% aminoacid identity with the stalk domain of the H1 vaccine strain), which all belong to the group 1 haemagglutinins but are phylogenetically distant to H1 (figure 1C), were used to determine antibody breadth. Titres are expressed as ELISA units (EU) per mL and were calculated on the basis of an internal standard to which units were arbitrarily assigned.

ELISpots were done on peripheral blood mono-nuclear cells (PBMCs) collected 7 days after each vaccination (study days 8 and 92) for plasmablast analysis and 28 days after each vaccination for memory B-cell analysis (study days 29 and 113). For memory B-cell responses, samples were also collected on study days 1 and 85 and analysed to assess cell counts before vaccination. For these assays, PBMCs were added to blocked ELISpot plates for 16 h overnight in an incubator set at 37°C with 5% CO_2_. After the overnight incubation, plates were washed and incubated with anti-IgG-biotin or anti-IgA-biotin (Southern Biotechnology, Birmingham, UK) for 1–2 h. After secondary antibody incubation, plates were washed and incubated with streptavidin-alkaline phosphatase (Southern Biotechnology) for 1–2 h. Plates were washed and developed with nitro blue tetrazolium/5-bromo-4-chloro-3-indolyl phosphate (Thermo Fisher Scientific, Waltham, USA) for 2–10 min, and reactions were then stopped by washing plates with distilled water and allowing them to dry overnight before counting. For memory B-cell analysis but not plasmablast analysis, to induce memory B-cell differentiation into antibody-secreting cells, 1 × 10^6^ PBMCs were stimulated with 10 ng/mL lectin pokeweed mitogen (Sigma-Aldrich, St Louis, USA), 1:100 000 diluted protein A from *Staphylococcus aureus*, Cowan Strain (Sigma-Aldrich, St Louis, USA), and 6 mg/mL CpG (Invitrogen, Carlsbad, USA) in an incubator at 37°C with 5% CO_2_ for 5 days. After stimulation, cells were counted and added to the ELISpot plates. Images were captured with Immunocapture 6.4 software, and spots were manually counted. Analyses were done using chimeric H6/1 and Cal09 H1 haemagglutinin recombinant proteins as antigens. Cell counts were expressed as spot-forming units per 1 × 10^6^ PBMCs; the limit of detection was four spot-forming units.

### Outcomes

The primary outcomes were reactogenicity and safety of the vaccination regimens. The secondary outcome was immunogenicity, including serum IgG and IgA to the H1 haemagglutinin stalk domain, serum neutralising antibodies to the H1 haemagglutinin stalk domain, antibody-dependent cellular cytotoxicity to the H1 haemagglutinin stalk domain, and saliva IgG and IgA and secretory IgA to the H1 haemagglutinin stalk domain 28 days after the prime and boost immunisations. Additionally, as a secondary outcome, we measured the breadth of the immune response (against H2, H9, H18 recombinant haemagglutinins, pandemic H1N1, and avian-swine H1N1 and H5N8) and its duration. Other secondary outcomes included the IgG response to N1 neuraminidase and H3 haemagglutinin; cell-mediated immune responses; the protective effect of human serum in a murine passive transfer-challenge study; and haemagglutination inhibition titres against chimeric H5/1N1, chimeric H8/1N1, chimeric H6/1N5, and pandemic H1N1.

This pre-planned interim analysis reports ELISA, B-cell data, and aggregated safety data up to day 113. The remaining data is expected to become available with the full study report at the end of 2019.

### Statistical analysis

Immunogenicity objectives included description of seroresponse rates and antibody geometric mean titres. Sample sizes of 14 or more evaluable participants per group provided more than 80% power for between-group comparisons of geometric mean titres using the Student’s *t* test on the log_10_ scale, assuming geometric mean titre ratios of greater than three, with a one-sided type I error level of 0·025 and assuming a SD of 0·4 in the log_10_ titres (based on previously published data).^29^ Only descriptive statistics were used for this interim analysis. We calculated seroresponse rates with two-sided 95% exact CIs, and pairing geometric mean titres and fold changes with two-sided 95% CIs based on the *t* distribution for the mean of the log-transformed data, followed by reversal of the log transformation. Because no specific threshold for these assays is known to be linked with protection, seroresponses were defined arbitrarily as four times or greater increases in titres as a descriptive measure. Immunogenicity was assessed in the per-protocol population, which included all participants without major deviations, including those considered likely to affect the immune response. Blinded safety analysis was done on all participants that received at least one vaccination. Statistical analysis was done with SAS software version 9.4.

This trial is registered with ClinicalTrials.gov, number NCT03300050.

### Role of the funding source

The funder of the study had no role in study design, data collection, data analysis, data interpretation, or writing of the report. The corresponding author had full access to all the data in the study and had final responsibility for the decision to submit for publication.

## Results

**Figure 2:**
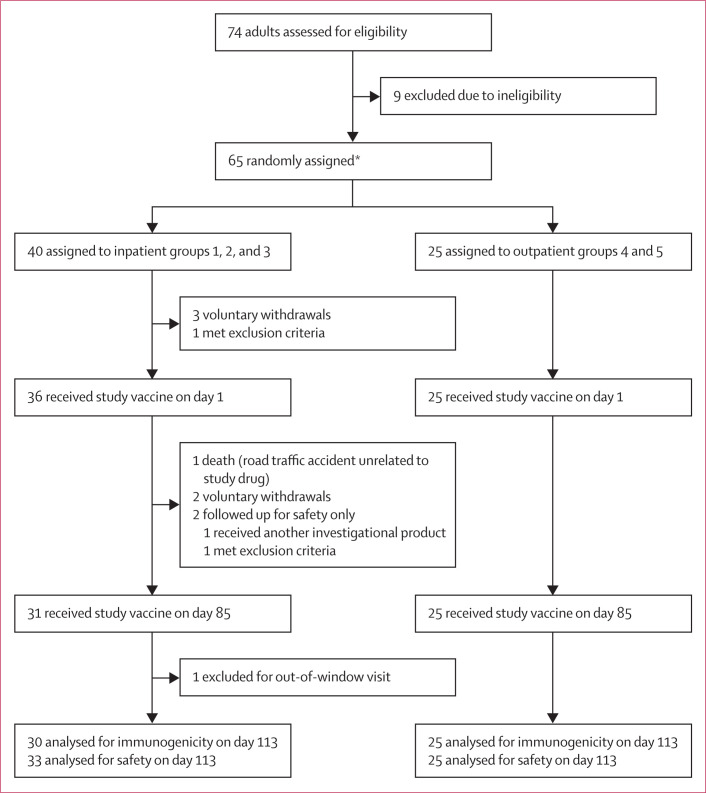
Trial profile Randomisation into inpatient (LAIV8-IIV5/AS03, LAIV8-IIV5, and sterile saline placebo control) and outpatient (IIV8/AS03-IIV5/AS03 and phosphate buffered saline placebo control) groups is shown. ^*^66 randomly assigned, with one ineligible participant included in error and excluded after randomisation.

Between Oct 10, 2017, and Nov 27, 2017, 65 participants were enrolled and randomly assigned (figure 2). Baseline demographic information (aggregated) is provided in the appendix (pp 1–2). 20 participants were randomly assigned to the LAIV8-IIV5/AS03 group, 15 to the LAIV8-IIV5 group, 15 to the IIV8/AS03-IIV5/AS03 group, ten to the PBS placebo group, and five to the sterile saline placebo group. Owing to the small group size and participant dropouts in the sterile saline group group (final group size was three), the results are not reported here.

For the interim analysis, safety results were blinded to study group (ie, disaggregated safety data were not available). One serious adverse event, a fatal motor vehicle accident that was unrelated to study vaccine, was reported. Within 28 days of any vaccination, 54 unsolicited adverse events were reported in 29 (48%) of the 61 participants. Of those, 35 were graded mild in 15 (25%) participants, 16 were graded moderate in 12 (20%) participants, and three were graded severe in two (3%) participants. Of the eight unsolicited adverse events reported as related to study vaccines, none were graded as severe, three were graded as moderate, and the remainder as mild. Medically attended adverse events were reported for 13 (21%) of 61 participants. Solicited local adverse events were reported in 12 (48%) of 25 outpatient participants and solicited systemic adverse events in 14 (56%) following prime immunisation with intramuscular study product. Following booster immunisation, solicited local adverse events were reported in 18 (32%) of 56 participants and solicited systemic adverse events in 21 (38%). Solicited local adverse events were reported in 12 (33%) of 36 participants and solicited systemic adverse events in 22 (61%) participants following intranasal study product administration. No potential immune-mediated diseases were reported, and no influenza A infections were confirmed in nine reported influenza-like illnesses.

None of the 36 inpatient participants in containment had a nasal or oropharyngeal swab positive for influenza A virus RNA by real-time PCR the day before administration of the chimeric H8/1N1 LAIV or placebo; daily swabs were positive in 11 participants on day 2 after administration, two on day 3, none on day 4, three on day 5, and none on day 6. Cycle threshold values were generally close to the cutoff value, indicating low amounts of viral nucleic acid. In Madin Darby canine kidney cell culture, aliquots from none of the 36 inpatient participants resulted in cytopathic effects or stained positive with chimeric H8/1 monoclonal antibody.

**Figure 3:**
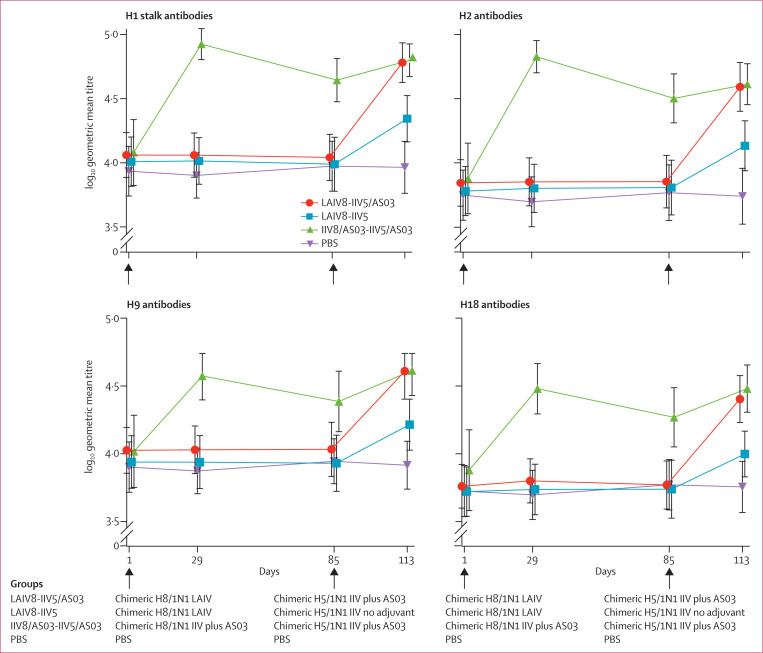
Titres of antibodies targeting the H1 stalk domain and heterosubtypic group 1 haemagglutinins Geometric mean ELISA antibody titres (ELISA units per mL) are plotted on the y axis (log_10_) for the timepoints indicated on the x axis. Error bars show the upper and lower limits of the 95% CIs. Vaccination timepoints for the LAIV8-IIV5/AS03, LAIV8-IIV5, IIV8/AS03-IIV5/AS03, and PBS groups are indicated below the x-axis. Group sizes are 19 for the LAIV8-IIV5/AS03 group, 14 for the LAIV8-IIV5 group, 15 for the IIV8/AS03-IIV5/AS03 group, and ten for the PBS group. IIV=inactivated influenza vaccine. LAIV=live-attenuated influenza vaccine. PBS=phosphate-buffered saline.

All study participants had pre-existing H1 stalk baseline titres on day 1 with geometric mean titres ranging from 8 615 to 12 028 EU (figure 3), meaning that all study participants were primed. After the first vaccine dose, no increase in anti-H1 stalk antibody titres was observed for the LAIV8-IIV5/AS03, LAIV8-IIV5, and placebo groups. By contrast, the IIV8/AS03-IIV5/AS03 group had a seven times increase (95% CI 4·3–11·4) in anti-stalk antibody titres to a geometric mean titre of 84 207 EU (63 756–111 219). In this group, 80·0% (51·9–95·7) of participants had an increase in titres of four times or higher (table) and 33% (11·8–61·6) had at least a ten times increase over baseline.

**Table:** Frequency of seropositivity at baseline and seroresponses on days 29 and 113 for the H1 stalk domain and heterosubtypic group 1 Haemagglutinins

	H1 stalk	H2	H9	H18
**Baseline (before vaccination; seropositive)**				
LAIV8-IIV5/AS03	19/19, 100·0% (82·4–100·0)	19/19, 100·0% (82·4–100·0)	19/19, 100·0% (82·4–100·0)	19/19, 100·0% (82·4–100·0)
LAIV8-IIV5	14/14, 100·0% (76·8–100·0)	14/14, 100·0% (76·8–100·0)	14/14, 100·0% (76·8–100·0)	14/14, 100·0% (76·8–100·0)
IIV8/AS03-IIV5/AS03	15/15, 100·0% (78·2–100·0)	15/15, 100·0% (78·2–100·0)	15/15, 100·0% (78·2–100·0)	15/15, 100·0% (78·2–100·0)
PBS	10/10, 100·0% (69·2–100·0)	10/10, 100·0% (69·2–100·0)	10/10, 100·0% (69·2–100·0)	10/10, 100·0% (69·2–100·0)
**Day 29 (28 days after prime dose; seroresponse)**				
LAIV8-IIV5/AS03	0/18, 0·0% (0·0–18·5)	0/18, 0·0% (0·0–18·5)	0/18, 0·0% (0·0–18·5)	0/18, 0·0% (0·0–18·5)
LAIV8-IIV5	0/14, 0·0% (0·0–23·2)	0/14, 0·0% (0·0–23·2)	0/14, 0·0% (0·0–23·2)	0/14, 0·0% (0·0–23·2)
IIV8/AS03-IIV5/AS03	12/15, 80·0% (51·9–95·7)	12/15, 80·0% (51·9–95·7)	6/15, 40·0% (16·3–67·7)	6/15, 40·0% (16·3–67·7)
PBS	0/10, 0·0% (0·0–30·8)	0/10, 0·0% (0·0–30·8)	0/10, 0·0% (0·0–30·8)	0/10, 0·0% (0·0–30·8)
**Day 113 (28 days after booster dose; seroresponse)**				
LAIV8-IIV5/AS03	11/15, 73·3% (44·9–92·2)	10/15, 66·7% (38·4–88·2)	5/15, 33·3% (11·8–61·6)	7/15, 46·7% (21·3–73·4)
LAIV8-IIV5	2/13, 15·4% (1·9–45·4)	1/13, 7·7% (0·2–36·0)	1/13, 7·7% (0·2–36·0)	2/13, 15·4% (1·9–45·4)
IIV8/AS03-IIV5/AS03	8/14, 57·1% (28·9–82·3)	10/14, 71·4% (41·9–91·6)	7/14, 50·0% (23·0–77·0)	7/14, 50·0% (23·0–77·0)
PBS	0/10, 0·0% (0·0–30·8)	0/10, 0·0% (0·0–30·8)	0/10, 0·0% (0·0–30·8)	0/10, 0·0% (0·0–30·8)

Values are n/N, % (95% CI). Participants were deemed seropositive at baseline if they had a positive ELISA value. Seroresponse was defined as four times or higher increases over baseline in antibodies. IIV=inactivated influenza vaccine. LAIV=live-attenuated influenza vaccine. PBS=phosphate buffered saline.

Serum anti-H1 stalk IgG antibody titres for the LAIV8-IIV5/AS03, LAIV8-IIV5, and placebo groups remained similar to baseline up to study day 85 (figure 3). Antibody titres for the IIV8/AS03-IIV5/AS03 group declined 2·0 times (95% CI 1·6–2·6) from day 29 to day 85 (day 85 geometric mean titre 44 093 EU/mL [29 942–64 931]). At day 113, 28 days after the booster vaccination, the LAIV8-IIV5/AS03 group had a 5·6 times (3·6–8·6) increase in anti-stalk antibody titres over baseline. A weaker induction of 2·2 times (1·6–3·2) was observed for the LAIV8-IIV5 group. The stalk antibody titres for IIV8/AS03-IIV5/AS03 group increased again from day 85 by 1·4 times (1·2–1·7; to 4·9 times [95% CI 3·2–7·5] over baseline). No changes were observed in the PBS placebo group. After the booster vaccination, the proportion of participants with a at least a four times increase above baseline was 73·3% (44·9–92·2) for the LAIV8-IIV5/AS03 group, 15·4% (1·9–45·4) for the LAIV8-IIV5 group, and 57·1% (28·9–82·3) for the IIV8/AS03-IIV5/AS03 group (table). A ten times or greater increase was achieved in 20·0% (4·3–48·1) of the LAIV8-IIV5/AS03 group, in 14·3% (1·8–42·8) of the IIV8/AS03-IIV5/AS03 group, and in no patients in the LAIV8-IIV5 group.

At baseline, geometric mean titres of anti-H2 antibodies were between 5 597 and 7 554 (figure 3). The results for induction of anti-H2 antibodies were similar to the results for induction of anti-H1 stalk antibodies. The IIV8/AS03-IIV5/AS03 group had an 8·9 times (5·2–15·1) increase in anti-H2 antibody titres after the first vaccination (geometric mean titre 67 191 [50 322–89 715]), a 2·2 times (1·7–2·9) decrease from this peak between day 29 and the pre-boost timepoint, and then an increase to a geometric mean titre of 41 005 (28 395–59 213) after the booster vaccination (day 113; figure 3B). As observed for the anti-H1 stalk antibody titres, no response was detected after the first vaccination for the LAIV8-IIV5/AS03 and LAIV8-IIV5 groups (day 29). Following the booster vaccination, the LAIV8-IIV5/AS03 group had a 5·8 times (3·8–8·8) increase over baseline, and a 2·2 times (1·7–2·9) increase over baseline was reported for the LAIV8-IIV5 group at day 113 (figure 3B). No induction of anti-H2 antibodies was detected for the placebo group. Similar antibody induction patterns for the respective vaccine groups were also detected for the more distant H9 and H18 subtypes (figure 3).

**Figure 4:**
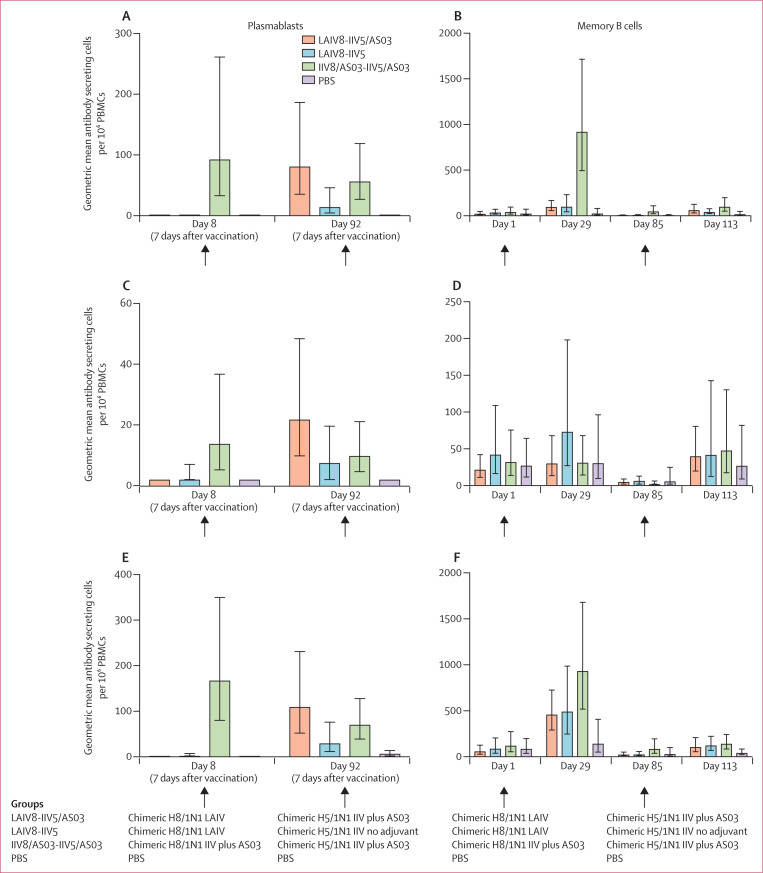
Plasmablast and memory B-cell responses to the H1 stalk and wild-type H1 haemagglutinins The error bars indicate the upper and lower limits of the 95% CIs. The lower limit of detection was four spot forming units per 10^6^ PBMCs. Plasmablasts were tested for H1 stalk-specific IgG (A), IgA (C), and Cal09 H1 IgA plus IgG (E) secretion on days 8 and 92 (7 days after vaccination). Memory B cells were tested for H1 stalk-specific IgG (B), IgA (D), and Cal09 H1 IgA plus IgG (F) secretion on days 1, 29, 85, and 113 (vaccination timepoints and 4-week post-vaccination timepoints). Group sizes are 19 for the LAIV8-IIV5/AS03 group, 14 for the LAIV8-IIV5 group, 15 for the IIV8/AS03-IIV5/AS03 group, and ten in the PBS group. IIV=inactivated influenza vaccine. LAIV=live-attenuated influenza vaccine. PBMC=peripheral blood mononuclear cells. PBS=phosphate-buffered saline.

A plasmablast IgG stalk response to the first vaccine dose was induced only in the IIV8/AS03-IIV5/AS03 group, with a geometric mean cell count at 7 days after vaccination of 92·7 spot-forming units per 10^6^ PBMCs (95% CI 32·9–261·1) compared with a geometric mean cell count below the lower limit of detection of four spotforming units per 10^6^ PBMCs in all other groups (figure 4A). After the booster dose, geometric mean counts of plasmablasts secreting H1 stalk IgG (in spot-forming units per 10^6^ PMBCs) were 81·1 (35·3–186·5) in the LAIV8-IIV5/AS03 group, 14·4 (4·5–46·0) in the LAIV8-IIV5 group, 56·5 (26·9–118·7) in the IIV8/AS03-IIV5/AS03 group, and below the lower limit of detection in the PBS control group.

The IgA plasmablast response was weaker than the IgG plasmablast response and again detectable only for the IIV8/AS03-IIV5/AS03 group after the first vaccination (13·8 spot-forming units per 10^6^ PMBCs [95% CI 5·2–36·7]; figure 4C). Booster vaccination elicited a response in all three vaccine groups (21·8 [9·8–48·4] spot-forming units per 10^6^ PBMCs in the LAIV8-IIV5/AS03 group, 7·5 [<4·0–19·6] in the LAIV8-IIV5 group, and 9·8 [4·6–211] in the IIV8/AS03-IIV5/AS03 group) but not in the placebo group (figure 4C). For wild-type Cal09 H1 haemagglutinin, we measured IgG-secreting and IgA-secreting cells together, and the magnitude of response resembled the IgG plasmablast response to the H1 stalk domain (figure 4E).

All groups showed baseline IgG and IgA memory B-cell reactivity to the haemagglutinin stalk before vaccination (figure 4B). Subsets of memory B cells with IgG specificities to the stalk domain expanded 28 days after prime in all vaccine groups, with the greatest increase in the IIV8/AS03-IIV5/AS03 group (figure 4B). Increases for LAIV8-IIV5/AS03 and LAIV8-IIV5 groups were lower than for the IIV8/AS03-IIV5/AS03 group but higher than the fluctuations measured in the PBS control group. At day 85 (before boost), levels of memory B cells were similar to placebo in all vaccine groups except in the IIV8/AS03-IIV5/AS03 group, although the CI does overlap with baseline. The number of memory B cells in the vaccine groups increased slightly above baseline after boost (day 113), but the increase was not comparable to the strong response seen in the IIV8/AS03-IIV5/AS03 group at 28 days after the first vaccination and, again, the CIs overlap with baseline. Very little response was observed for stalk-reactive IgA memory B cells (figure 4D). Memory B-cell counts (IgG and IgA combined) to the wild type Cal09 H1 of pandemic H1N1 were generally higher than for H1 stalk IgG at baseline but also followed the pattern seen for the anti-stalk IgG memory B-cell response (figure 4F).

## Discussion

We report the interim results of a phase 1 study with the first rationally designed universal influenza virus vaccines targeting the haemagglutinin stalk domain. We observed that a single vaccination with an adjuvanted chimeric haemagglutinin-based IIV, but not with a nonadjuvanted LAIV, induced high serum IgG stalk-reactive antibody titres. This initial response to the IIV was detected in a vast majority of study participants and was heterosubtypic, with confirmed cross-reactivity to H2, H9, and H18 haemagglutinins. Given this breadth and the phylogenetic distance between H1, H2, H9, and H18, the induced response would likely cross-react to all other members of the group 1 haemagglutinins. Additionally, on the basis of the blinded study group, the vaccine regimens were tolerable and no safety concerns were observed.

We previously found that high concentrations of stalk-reactive antibodies in human serum protected mice from lethal virus challenge in a passive serum transfer study.^30^ Additionally, stalk-reactive serum antibodies were correlated with reduced virus shedding and symptom numbers in a human challenge model (but not duration or severity of symptoms),^31^ and were shown by Ng and colleagues^32^ to independently correlate with protection from natural pandemic H1N1 infection (as defined by PCR positivity) and disease in children and adults. Therefore, a single dose of chimeric, haemagglutinin-based, adjuvanted IIV might induce protective titres against all group 1 haemagglutinin-expressing viruses, making it an excellent candidate for development as a group 1 pandemic vaccine.

Although the induction of anti-stalk serum IgG and peripheral blood plasmablast and memory B-cell responses by the IIV formulation was obvious, it is less clear whether an immune response was induced with a chimeric haemagglutinin-based LAIV because no serum IgG response was detected after the first vaccination. This lack of response might be due to insufficient replication of the vaccine virus or an inadequate dose of the LAIV. However, seasonal LAIVs administered to adults are known to elicit a weak serum antibody response but potent mucosal immunity.^33,34^ It is, therefore, likely that the lack of a serum IgG response is, at least partly, a reflection of the inherent properties of the vaccine platform. Additionally, pre-pandemic LAIVs, including vaccines based on H2, H5, and H6, induced no or very weak responses in adults^35–37^ but primed efficiently for strong and broad responses once the same individuals were boosted with an IIV of the same subtype.^21^

The booster immunisation with chimeric H5/1N1 IIV induced an immune response in most vaccinees, including those who had been primed with LAIV or IIV. Both the LAIV8-IIV5/AS03 and the IIV8/AS03-IIV5/AS03 groups showed almost the same titres after the booster vaccination, suggesting little priming by the initial LAIV dose in terms of serum IgG titres. Adults are typically already primed for the stalk domain, as can be seen by baseline serum IgG titres and baseline memory B-cell levels. The finding of high baseline titres agrees with earlier studies that found low but widely prevalent pre-existing immunity against the H1 stalk domain in adults and even in children, and these titres are usually induced by natural infection.^8,9,38^ Therefore, one potential explanation is that the LAIV prime was minimal relative to the already existing priming for serum IgG. However, it is possible that the LAIV prime leads to higher quality serum antibodies (eg, higher affinity or better effector functions), a more sustained antibody response, or higher titres of mucosal antibodies than priming with the IIV. This question remains to be explored as part of the extended immune analysis, which will assess mucosal immune responses, including mucosal IgG and IgA and secretory IgA, and peripheral blood T-cell responses. The true strength of this clinical trial might reside in yet to be documented mucosal immune responses, so the LAIV approach should not be discounted prematurely.

The LAIV8-IIV5 regimen resulted in markedly lower titres than the LAIV8-IIV5/AS03 regimen, suggesting that an adjuvant is needed for optimal induction of stalk-reactive antibodies in adults, which is in agreement with preclinical findings in mice and ferrets.^18,19^ Although the initial vaccination with adjuvanted chimeric H8/1N1 IIV induced a strong immune response to the stalk, the booster with chimeric H5/1N1 IIV was not as effective, and post-boost antibody concentrations did not exceed those achieved after the first dose (for the IIV8/AS03-IIV5/AS03 group). Potential explanations for this finding are that the high titres of stalk-reactive antibodies induced by the first vaccination (IIV8/AS03) in this group masked or cleared the antigen and blunted the immune response. Alternatively, it could be that a response ceiling was already reached. A third explanation is that the first antigen (chimeric H8/1) was more immunogenic than the second (chimeric H8/1), although this was not the case in pre-clinical experiments in mouse models.^27^

With regard to the dynamics of memory B cells, the IIV8/AS03 prime induced a strong memory response as expected, but this did not occur in the other groups after the second dose, despite boosts in plasmablasts and antibodies. This finding is notable and could be due to several reasons—eg, the peak of the memory B cell response might have been missed or memory B-cells might have migrated to tissues reducing their numbers in peripheral blood. It could also be that the anti-stalk memory B-cells might have already peaked with the prime immunisation and cannot be boosted further or prime and boost might have been too close together diminishing the generation of more anti-stalk memory-B cells through epitope masking.

This interim analysis has several limitations. The data available are limited to the timepoints and assays of the per-protocol interim analysis. It includes only two of the five key immunological readouts of the study and none of the timepoints that would allow analysis of antibody persistence. Additional data will become available for the full per-protocol analysis and will shed more light on the questions discussed above.

In summary, the interim analysis of this phase 1 trial suggests that a chimeric haemagglutinin-based adjuvanted IIV induces high titres of anti-stalk antibodies that strongly cross-react with heterosubtypic group 1 haemagglutinins, supporting further development of this candidate vaccine as part of a universal influenza virus vaccine strategy.

### Contributors

AN, RN, FBS, JF, PCW, AS, MVdW, CC, RAA, MEE, RH, WS, RW, HW, TJW, BLI, AG-S, PP, and FK participated in the conceptualisation of the immunogens and the clinical study design. KNB, YC, MD HLD, DF, MG, JJG, CH, LY-LL, MM, AKEP, DGS, CTS, VS, and MET collected data. DIB, JG, AN, RN, FB-S, JF, PCW, EBW, CG, CH, MM, BLI, AG-S, and FK analysed and curated data. DIB, JG, EBW, KNB, MD, DF, MG, and VS were study investigators. JF, RW, MVdW, and AS were responsible for study resources. All authors participated in writing and reviewing of the manuscript.

### Declaration of interests

The Icahn School of Medicine at Mount Sinai (ISMMS) has issued patents and filed patent applications covering the use of chimeric haemagglutinin antigens as vaccines. RN, AG-S, PP, and FK, are named as inventors on these patents and applications. The ISMMS has granted GlaxoSmithKline (GSK) certain license rights to these patents and patent applications. The ISMMS and the inventors have received payments as consideration for these rights. The laboratories of AG-S, PP, and FK are also engaged in a research programme that is funded by GSK. MVdW, CC, and MEE are employees of GSK. BLI was a GSK employee until 2017 and is also named as inventor on patent applications covering chimeric haemagglutinin antigens. All other authors declare no competing interests.

### Data sharing

Anonymised group-level participant data can be requested for further research.
