# Supplementary material for: Immunogenicity of chimeric haemagglutinin-based, universal influenza virus vaccine candidates: interim results of a randomised, placebo-controlled, phase 1 clinical trial
Source: Lancet Infect Dis. 2019 Oct 17;20(1):80–91. doi: 10.1016/S1473-3099(19)30393-7 (PMC6928577; doi:10.1016/S1473-3099(19)30393-7)
Supplement: Supplementary file 1 [file LID-2019-S1473-3099(19)30393-7-s1.pdf]

THE LANCET Infectious Diseases

Supplementary webappendix

This webappendix formed part of the original submission and has been peer reviewed. We post it as supplied by the authors.

Supplement to: Bernstein DI, Guptill J, Naficy A, et al. Immunogenicity of chimeric haemagglutinin-based, universal influenza virus vaccine candidates: interim results of a randomised, placebo-controlled, phase 1 clinical trial. *Lancet Infect Dis* 2019; published online Oct 17. https://doi.org/10.1016/S1473-3099(19)30393-7.

Suppl. Table 1. Categorical Demographics and Baseline Characteristics - All Enrolled Subjects

		CCHMC (N=26)		DCRI[a] (N=39)		All Subjects (N=65)	
**Demographic Category**	**Characteristic**	**n**	**%**	**n**	**%**	**n**	**%**
Gender	Female	18	69.2%	22	56.4%	40	61.5%
	Male	8	30.8%	17	43.6%	25	38.5%
Ethnicity	Hispanic or Latino	1	3.8%	2	5.1%	3	4.6%
	Not Hispanic or Latino	25	96.2%	37	94.9%	62	95.4%
	Not Reported	0	0%	0	0%	0	0%
	Unknown	0	0%	0	0%	0	0%
Race	American Indian/ Alaskan Native	0	0%	0	0%	0	0%
	Asian	0	0%	1	2.6%	1	1.5%
	Native Hawaiian or Other Pacific Islander	0	0%	0	0%	0	0%
	Black or African American	23	88.5%	26	66.7%	49	75.4%
	White	2	7.7%	11	28.2%	13	20.0%
	Multi-Racial	1	3.8%	0	0%	1	1.5%
	Unknown	0	0%	1	2.6%	1	1.5%

Note: N = number of enrolled subjects.

[a] Excludes one subject who was randomized but replaced prior to receiving study treatment

CCHMC=

DCRI=

Suppl. Table 2. Continuous Demographics and Baseline Characteristics - All Enrolled Subjects

		CCHMC (N=26)	DCRI[a] (N=39)	Subjects (N=65)
Age (years)	Mean	27.6	30.0	29.0
	Standard Deviation	5.5	5.2	5.4
	Median	27.0	31.0	29.0
	Minimum	19	18	18
	Maximum	38	37	38
Height (cm)	Mean	170.1	169.6	169.8
	Standard Deviation	10.2	9.4	9.6
	Median	168.8	168.0	168.5
	Minimum	155	155	155
	Maximum	195	188	195
Weight (kg)	Mean	79.2	80.5	80.0
	Standard Deviation	17.2	16.2	16.5
	Median	78.9	81.6	81.1
	Minimum	51	47	47
	Maximum	120	118	120

Note: N = number of enrolled subjects.
[a] Excludes one subject who was randomized but replaced prior to receiving study treatment.
CCHMC=
DCRI=
